# Synthesis of an antiviral drug precursor from chitin using a saprophyte as a whole-cell catalyst

**DOI:** 10.1186/1475-2859-10-102

**Published:** 2011-12-05

**Authors:** Matthias G Steiger, Astrid R Mach-Aigner, Rita Gorsche, Erwin E Rosenberg, Marko D Mihovilovic, Robert L Mach

**Affiliations:** 1 Gene Technology and Applied Biochemistry, Institute of Chemical Engineering, Vienna University of Technology, Gumpendorfer Str. 1a, A-1060 Wien, Austria; 2 Instrumental Analytical Chemistry, Institute of Chemical Technologies and Analytics, Vienna University of Technology, Getreidemarkt 9/164, A-1060 Wien, Austria; 3 Institute of Applied Synthetic Chemistry, Vienna University of Technology, Getreidemarkt 9/163, A-1060 Wien, Austria; 4 Austrian Centre of Industrial Biotechnology, Muthgasse 107, 1190 Vienna, Austria

## Abstract

**Background:**

Recent incidents, such as the SARS and influenza epidemics, have highlighted the need for readily available antiviral drugs. One important precursor currently used for the production of Relenza, an antiviral product from GlaxoSmithKline, is N-acetylneuraminic acid (NeuNAc). This substance has a considerably high market price despite efforts to develop cost-reducing (biotechnological) production processes. *Hypocrea jecorina *(*Trichoderma reesei*) is a saprophyte noted for its abundant secretion of hydrolytic enzymes and its potential to degrade chitin to its monomer N-acetylglucosamine (GlcNAc). Chitin is considered the second most abundant biomass available on earth and therefore an attractive raw material.

**Results:**

In this study, we introduced two enzymes from bacterial origin into *Hypocrea*, which convert GlcNAc into NeuNAc via N-acetylmannosamine. This enabled the fungus to produce NeuNAc from the cheap starting material chitin in liquid culture. Furthermore, we expressed the two recombinant enzymes as GST-fusion proteins and developed an enzyme assay for monitoring their enzymatic functionality. Finally, we demonstrated that *Hypocrea *does not metabolize NeuNAc and that no NeuNAc-uptake by the fungus occurs, which are important prerequisites for a potential production strategy.

**Conclusions:**

This study is a proof of concept for the possibility to engineer in a filamentous fungus a bacterial enzyme cascade, which is fully functional. Furthermore, it provides the basis for the development of a process for NeuNAc production as well as a general prospective design for production processes that use saprophytes as whole-cell catalysts.

## Background

NeuNAc is the most prevalent exponent of sialic acids [1]. In mammals, sialic acids are usually found as terminal residues of glycol conjugates on the outermost cell surface. As a result of their location and their negative carboxylate functionality, sialic acids play important roles in mediating cellular recognition and adhesion processes [2] and in the infection cycles of severe viral diseases, such as influenza viruses A and B [3]. In these cases, *de novo-*synthesized viral particles attach to their respective sialic acids at the cell surface. Neuraminidase (sialidase) activity is needed for the propagation of the virus in the host. Consequently, sialic acid derivatives are successfully applied in the therapy of such virus-related diseases. One well-known product that functions as a neuraminidase inhibitor is Relenza. Its active pharmaceutical ingredient is Zanamivir, which is a direct derivative of the NeuNAc precursor [4].

Traditionally, NeuNAc is prepared through extraction from natural sources, such as bird nests, milk, or eggs [5], through the hydrolysis of colominic acid (a homopolymer of NeuNAc) in a culture broth of *Escherichia coli *K1 [6], or through chemical synthesis [7]. Methods for NeuNAc production have included a chemo-enzymatic process [8,9], a two-enzyme reaction process [10,11], a biotransformation process using *E. coli *[12], and an *E. coli *whole-cell system [13]. However, the requirement for ATP or an excess of pyruvate and the subsequent expensive downstream processing has kept the costs of NeuNAc production considerably high (current market price is $100/g).

Chitin is considered the second most abundant biomass available on earth [14]. The estimated annual biosynthesis of chitin is more than 10^11 ^tons in marine waters alone [15]. Unlike cellulose, the other dominant biopolymer, chitin can serve as a source for both carbon and nitrogen (C:N = 8:1) [16]. This property suggests that chitin is an optimal resource for the production of NeuNAc (C:N = 11:1) because no additional nitrogen would need to be applied as it would be if glucose or cellulose were used as raw material. Chitin is found in the exoskeletons of arthropods, such as crustaceans (including crab, lobster, and shrimp) and insects (including ants and beetles), the cell walls of fungi, the radula of mollusks, and the beaks of cephalopods (including squid and octopi). This polymer is composed of β-(1,4)-linked units of the amino sugar N-acetylglucosamine (GlcNAc) that is currently produced using hydrolysis of deproteinized and demineralized crustacean shells [17]. Chitinolytic enzymes from fungi of the genus *Hypocrea *have been extensively studied for decades [18]. More recently, the chitinolytic enzyme system of *H. jecorina *has been studied using genome-wide analysis [19,20]. Unlike their bacterial counterparts (e.g., *Serratia marcescens *[21]), *Hypocrea *chitinolytic preparations have a high ratio of exochitinase to endochitinase activity and almost exclusively release monomeric GlcNAc from chitin [22], which is another advantageous aspect of chitin compared to cellulose. Nevertheless, this raw material has not been adequately used. Therefore, the basic premise of this study was to exploit the potential of a saprophytic fungus to degrade the cheap biowaste chitin to its monomer GlcNAc and to further metabolize this product to NeuNAc.

## Results and Discussion

### Engineering a NeuNAc synthesis pathway into *Hypocrea*

**Figure 1:**
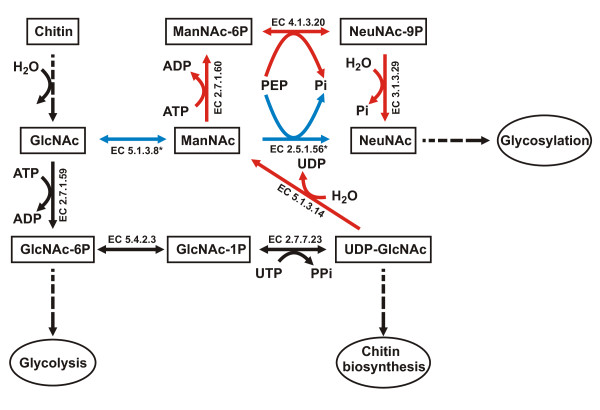
**An illustration of the NeuNAc synthesis-related pathways in microorganisms**. Boxes represent substances or metabolites, ellipses represent general biochemical processes, black solid lines represent enzymatic reactions in *H. jecorina*, black dashed lines represent pathways involving more than one enzymatic reaction in *H. jecorina*, red lines represent enzyme reactions not occurring in *H. jecorina*, and blue lines represent heterologously expressed bacterial enzymes. *EC numbers EC 5.1.3.8 (GlcNAc-2-epimerase) and EC 2.5.1.56 (NeuNAc synthase) refer to bacterial enzymes that were heterologously expressed using *Hypocrea *codon-optimized genes. EC 4.1.3.20, N-acylneuraminate-9-phosphate synthase; EC 3.1.3.29, N-acylneuraminate-9-phosphatase; EC 2.7.1.60, N-acylmannosamine kinase; EC 5.1.3.14, UDP-N-acetylglucosamine 2-epimerase; EC 5.4.2.3 Phosphoacetylglucosamine mutase; EC 2.7.7.23, UDP-N-GlcNAc diphosphorylase; EC2.7.1.59, N-acetylglucosamine kinase; GlcNAc, N-acetylglucosamine; ManNAc, N-acetylmannosamine; NeuNAc, N-acetylneuraminic acid; P, phosphate group.

The biosynthesis of NeuNAc begins with the formation of N-acetylmannosamine (ManNAc) from GlcNAc or UDP-N-acetylglucosamine (UDP-GlcNAc). In mammals, ManNAc is then phosphorylated to give ManNAc-6-phosphate (ManNAc-6P). The second step involves the condensation of either ManNAc (in bacteria) or MacNAc-6P (in mammals) with phosphoenolpyruvate (PEP) to give NeuNAc or NeuNAc-9P, respectively. In mammals, NeuNAc-9P is then dephosphorylated to generate NeuNAc (see Figure 1). *Hypocrea *naturally degrades chitin almost exclusively to GlcNAc [22]. Therefore, the challenge was to engineer a pathway to convert GlcNAc to NeuNAc via ManNAc, which would enable the use of *Hypocrea *as a whole-cell catalyst.

Lee and coworkers found that whole-cell extracts of several photobacteria could convert GlcNAc to ManNAc [13]. Among them, *Anabaena *sp. CH1 exhibited the highest GlcNAc 2-epimerase activity; consequently, they cloned and characterized a gene encoding GlcNAc 2-epimerase from *Anabaena *sp. CH1 (E.C. 5.1.3.8), which was used in the present study as a *Hypocrea *codon-optimized gene. For the second step (the condensation of ManNAc to NeuNAc), the currently used enzyme-catalyzed processes use a lyase, which requires an excess of pyruvate. Use of this incurs high downstream processing costs. Therefore, we used the NeuNAc synthase (EC 2.5.1.56) from *Campylobacter jejuni *[23] in the *Hyprocrea *process. This enzymatic step entails the use of PEP instead of pyruvate, which in the intended *in vivo *process is supplied by the fungus, thereby leading to an irreversible and more efficient reaction towards NeuNAc [24]. Moreover, the need for an excess of pyruvate becomes obsolete, and the resulting downstream process is significantly simplified. Similar to the GlcNAc 2-epimerase, the coding sequence for the NeuNAc synthase was codon-optimized for the usage in *Hypocrea*. The synthetic pathway is presented in Figure 1. The complete nucleotide sequences for both genes encoding the recombinant enzymes, *tbage *and *tneub*, are shown in additional file 1.

### Metabolization or uptake of NeuNAc can not be observed in *Hypocrea*

**Figure 2:**
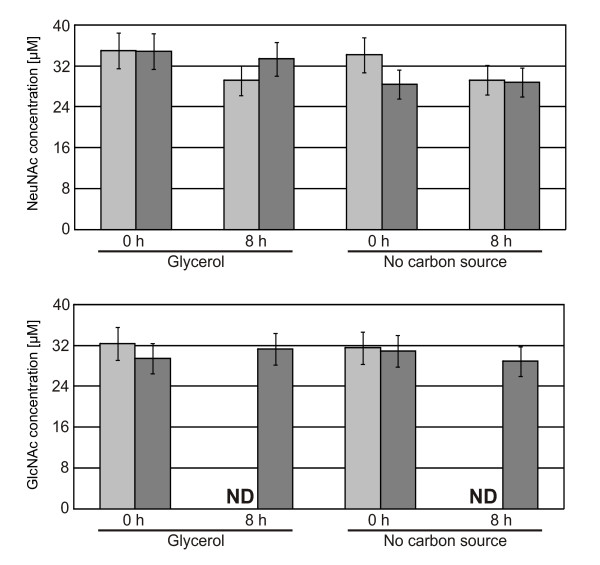
**The analysis of possible metabolization of NeuNAc in *H. jecorina***. The parental strain was pre-cultured on glycerol, and half of the mycelia were autoclaved. Living (light grey) and dead (dark grey) mycelia were transferred to MA media containing glycerol or MA media that lacked a carbon source, and NeuNAc (a) or GlcNAc (b) (as positive control) was added to both media. Samples were collected at 0 and 8 h. For NeuNAc analysis supernatants were derivatized using DMB befor analyses using HPLC. The presented values are the means of two biological duplicates that were derivatized in duplicate. Error bars indicate the standard deviations.

As the ability of the fungus to metabolize NeuNAc is an important issue, a possible uptake of NeuNAc by *H. jecorina *was investigated. Therefore, the fungus was pre-grown on glycerol in liquid culture, and half of the mycelium was autoclaved and half of it was harvested. The dead and living mycelia were transferred to glycerol-containing medium to study growth conditions or to medium without a carbon source to study resting cell conditions. NeuNAc was added to both media, and cultures were incubated for 8 h. Supernatants from all conditions were analyzed after incubation for 0 and 8 h by HPLC after derivatization using 1,2-diamino-4,5-methylenedioxybenzene dihydrochloride (DMB). Similar amounts of NeuNAc were present under all conditions regardless of whether the fungus was alive or dead (Figure 2a). This result indicates that NeuNAc uptake does not occur in *H. jecorina*. As a positive control experiment we did a similar experiment but instead of NeuNAc, GlcNAc was added to the media. As can be inferred from Figure 2b GlcNAc was completely consumed after eight hours under both growth and resting cell conditions when the mycelium was viable.

### Characterization of the recombinant *H. jecorina *strain

Recombinant *Hypocrea *strains were generated using protoplast transformation of *H. jecorina *QM9414. In the derived strains, the two *Hypocrea *codon-optimized genes (without GST-tag) were placed under the control of either the *H. jecorina *pyruvate kinase (*pki*) promoter, which is a strong constitutive promoter, or the *H. jecorina *xylanase 1 (*xyn1*) promoter, which is a strict shut-off system if an inducer (*e.g*. D-xylose) is missing. Such a system was used to avoid interference of the introduced recombinant pathway with cell wall biosynthesis and consequently, biomass formation. However, when comparing both promoter systems the strong *pki *promoter did not lead to decreased growth, diminished cell integrity or other adverse effects (data not shown). Therefore, we used strains in which both genes were under the control of the *pki *promoter for further studies as we observed a remarkably higher NeuNAc formation.

Transcriptional analysis of the recombinant *H. jecorina *strains was done by RT-qPCR to compare expression of both inserted genes using *sar1 *(SAR/ARF-type small GTPase) as a stable reference gene [25]. Furthermore, the copy numbers of both genes was measured by qPCR of genomic DNA using *pki *as a reference, which in the native *H. jecorina *genome is present as a single copy gene. Based on these analyses a strain (termed PEC/PSC1) was chosen for further investigations because it showed the highest equal expression of both inserted genes. This was confirmed by the finding that this strain bears two copies of each recombinant gene in the genome. These data were also supported using Southern blot analysis (data not shown).

### GlcNAc 2-epimerase and NeuNAc synthase are fully functional as GST-fusion proteins

Both recombinant enzymes were heterologously expressed as glutathione S-transferase (GST) fusion proteins in *E. coli*; the affinity chromatography purified proteins were used to confirm that their enzymatic capability was not altered by the codon usage adaptation and to provide a positive control for the enzymatic assays later on.

**Figure 3:**
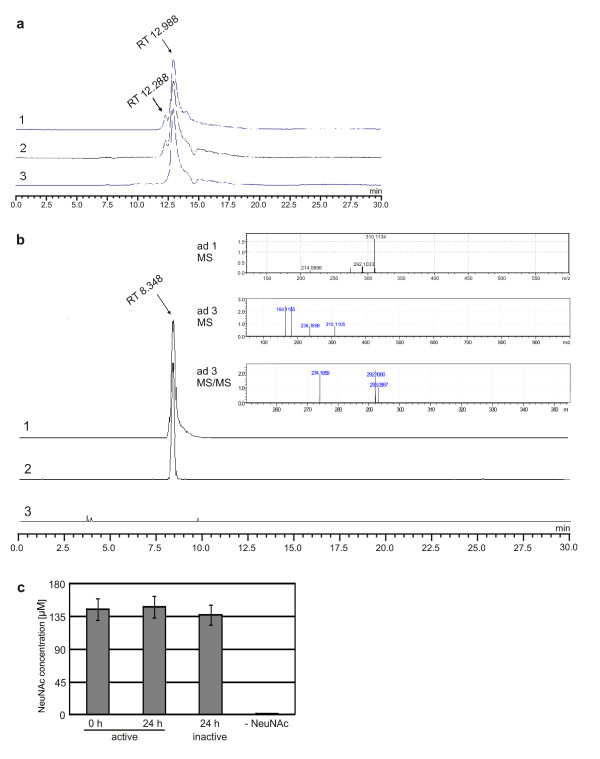
**NeuNAc synthesis *in vitro *in an enzymatic assay**. (a) EICs of the HPLC-MS analysis at 222.098 atomic mass units (amu) corresponding to the mass of the [GlcNAc+H]^+ ^ion and the [ManNAc +H]^+ ^ion. Retention times (RTs) of ManAc (12.288 min) and GlcNAc (12.988 min) are indicated. (1) Chromatogram of the *in vitro *assay using GST fusion proteins of GlcNAc-2-epimerase and NeuNAc synthase. (2) Chromatogram of the *in vitro *assay using a cell-free extract of the PEC/PSC1 strain. (3) Chromatogram of the *in vitro *assay using a cell-free extract of the parental strain. (b) EICs at 310.1134 amu, corresponding to the mass of the [NeuNAc+H]^+ ^ion. RT of NeuNAc (8.348 min) is indicated. (1) Chromatogram of the *in vitro *assay using GST fusion proteins of GlcNAc-2-epimerase and NeuNAc synthase. (2) Chromatogram of the *in vitro *assay using a cell-free extract of the PEC/PSC1 strain showing an 8-fold amplification compared to (1). (3) Chromatogram of a cell-free extract of the parental strain showing a 250-fold amplification compared to (1). (ad 1 MS) and (ad 2 MS) are MS spectra of chromatograms 1 and 2, respectively, at a RT of 8.348 min. (c) Cell-free extracts (active) of the PEC/PSC1 strain obtained from cultivation on chitin were mixed with NeuNAc and incubated for 0 and 24 h. A heat-inactivated cell-free extract was similarly treated. An active cell-free extract without NeuNAc (-NeuNAc) was also incubated, DMB-derivatized and analyzed. Values are means of biological duplicates derivatized in duplicate. Error bars indicate standard deviations.

To determine if the recombinant enzymes were functional, both GST fusion proteins were used in an enzymatic assay. The presence of GlcNAc and the formation of the intermediate product ManNAc and the final product NeuNAc were monitored using HPLC-MS analysis and results are presented in Figure 3. Application of the GST-fusion proteins of both enzymes in the *in vitro *assay led to the formation of ManNAc and NeuNAc demonstrating that the synthetic genes are expressed as functional proteins (Figure 3a1 and 3b1).

### NeuNAc synthesis *in vitro *by recombinant *H. jecorina *strains

According to the GST-fusion proteins, cell-free extracts of the recombinant *H. jecorina *strain PEC/PSC1 were applied in the enzymatic assay. The formation of ManNAc (Figure 3a2) and NeuNAc could be detected (Figure 3b2). This demonstrates that both enzymes are also fully functionally expressed in the recombinant *H. jecorina *strain PEC/PSC1. Neither ManNAc nor NeuNAc was detected using cell-free extracts from the parental strain in the assay (Figure 3a3 and 3b3), indicating that these pathways are normally not active in *Hypocrea*.

To investigate the stability of NeuNAc in cell-free extracts of the recombinant strain, according cell-free extracts obtained from the cultivation in a bioreactor on chitin (*vide infra*) were spiked with NeuNAc and incubated for 24 h. As a control, a heat-inactivated cell-free extract was similarly treated. Using HPLC analysis after derivatization with DMB, similar amounts of NeuNAc were detected in both extract preparations (Figure 3c), suggesting that components of the cell-free extract do not actively degrade NeuNAc. In addition, a similar amount of NeuNAc was measured in a NeuNAc-spiked cell-free extract of the recombinant strain that was not incubated, assuming that the 24-h incubation period at 30°C did not decrease the NeuNAc levels. As a final control, a cell-free extract without NeuNAc was also analyzed after a 24-h incubation period and, as expected, showed a lower amount of NeuNAc, which could only have resulted from its formation during the cultivation on chitin. In summary, we did not observe degradation of NeuNAc by *H. jecorina*. These data suggest that NeuNAc is not metabolized by the recombinant *Hypocrea *strain.

### NeuNAc synthesis *in vivo *by the recombinant *H. jecorina *strain

**Figure 4:**
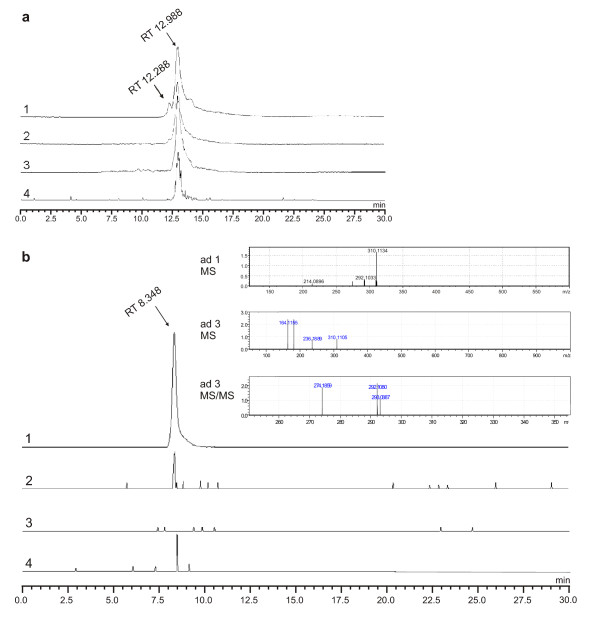
**NeuNAc synthesis *in vivo *in the *H. jecorina *PEC/PSC1 strain**. (a) EICs from the HPLC-MS analysis at 222.098 atomic mass units (amu) corresponding to the mass of the [GlcNAc+H]^+ ^and [ManNAc +H]^+ ^ions. Retention times (RTs) of ManAc (12.288 min) and GlcNAc (12.988 min) are indicated. (1) Chromatogram of the *in vitro *assay using GST fusion proteins of GlcNAc-2-epimerase and NeuNAc synthase. (2) Chromatogram of the cell-free extract of the PEC/PSC1 strain grown on GlcNAc. (3) Chromatogram of the cell-free extract of the parental strain grown on GlcNAc. (4) Chromatogram of the cell-free extract of the PEC/PSC1 strain grown on chitin. (b) EICs at 310.1134 amu corresponding to the mass of the [NeuNAc+H]^+ ^ion. RT of NeuNAc (8.348 min) is indicated. (1) Chromatogram of the *in vitro *assay using GST fusion proteins of GlcNAc-2-epimerase and NeuNAc synthase. (2) Chromatogram of the cell-free extract of the PEC/PSC1 strain grown on GlcNAc showing 25-fold amplification compared to (1). (3) Chromatogram of the cell-free extract of the parental strain showing a 250-fold amplification compared to (1). (4) Chromatogram of the cell-free extract of the PEC/PSC1 strain after cultivation on chitin for 90 h showing a 25-fold amplification compared to (1). (ad 1 MS) and (ad 4 MS) are the MS spectra of chromatograms 1 and 4, respectively, at RTs of 8.348 min. (ad 4 MS/MS) is the MS/MS spectrum of chromatogram 3 at a RT of 8.348 min.

We next addressed whether the recombinant *H. jecorina *strain had the ability to produce NeuNAc *in vivo*. To test this, the strain was grown on GlcNAc in shake flasks and cultivated on colloidal chitin in a bioreactor. Data on the corresponding cultivation monitoring are provided in additional file 2. As a positive control, an enzyme assay using the GST fusion proteins was again performed and resulted in the detection of ManNAc using HPLC-MS analysis as shown in Figure 4a1. Notably, the intermediate ManNAc was detected in the recombinant strain, regardless of the carbon source (Figure 4a2 und Figure 4a4), whereas the parental strain did not form ManNAc (Figure 4a3). In the parental strain, only the first metabolite, GlcNAc, was detected, and it was present because it was either directly used as a carbon source or formed by degradation of the biopolymer chitin due to the native chitinolytic activity of the fungus. The synthesis of the product NeuNAc was analyzed using HPLC-MS/MS analysis (Figure 4b). As a positive control, the reaction products (ManNAc, NeuNAc) generated by the use of the GST fusion proteins in an enzymatic assay are shown (Figure 4b1). Importantly, the recombinant *H. jecorina *strain formed NeuNAc using either carbon source, GlcNAc or chitin (Figure 4b2 and 4b4), whereas in the parental strain no formation of NeuNAc was detected (Figure 4b3). This analysis allowed us to estimate that 13 μg NeuNAc per g mycelium (dry weight) was formed in the recombinant strain. Thus, on its own, this would not be a competitive production process, but it does demonstrate the possibility for engineering a saprophyte and using it as a whole-cell catalyst that expresses a bacterial enzyme cascade. This method has enormous potential considering its use of a cheap starting material and the relatively simple, inexpensive cultivation of a fungus.

## Conclusions

Taken together, we successfully engineered *Hypocrea *in a way that this fungus now produces NeuNAc from the biopolymer chitin by employing its natural saprophytic activity in combination with the introduction of a bacterial enzyme cascade. Because human society will face severe bottlenecks in the supply of energy and in obtaining certain raw materials in the upcoming years, we hope that this study will highlight the potential advantages of biopolymers, such as chitin, and stimulate their efficient usage. Furthermore, we anticipate that such strategies will support efforts to create sustainable production processes.

## Methods

### Strains and cultivation conditions

The parental strain *H. jecorina *(*T. reesei *[26]) QM9414 (ATCC 26921) was maintained on malt extract (MEX) agar.

Mycelia for the enzymatic assay were cultivated in 3% (w/v) MEX medium using 10^8 ^conidia/L at 30°C.

Cultivation of *H*. *jecorina *on colloidal chitin was performed in a bench top bioreactor (Bioengineering, Wald, Switzerland) as previously described [27]. Briefly, 500 mL Mandels-Andreotti (MA) [28] medium containing 1% (w/v) colloidal chitin [29], 0.5% GlcNAc, and 0.1% (w/v) bacto peptone (Difco, Detroit, US) was inoculated with 10^8 ^conidia/L. Some drops glanapon (Becker, Wien, Austria) were added to the medium to avoid excessive foam formation. Cultivation was performed at 30°C temperature, pH 5, 0.3 vvm aeration rate, and 500 rpm agitation rate for 96 h. Each sample drawing was followed by a microscopic analysis for infection control. Culture supernatant and mycelia were separated by filtration through GF/F glass microfiber filters (Whatman, Brentford, UK). All strains (parental, recombinant) showed similar growth on rich media as well as MA medium.

### Plasmid construction

The synthetic gene *tbage *(for sequence see additional file 1) is based on the protein sequence of *Anabaena sp*. CH1 GlcNAc-2-epimerase (GenBank: ABG57042) and was reverse translated into a nucleotide sequence using the GeneOptimizer^® ^software (Geneart, Regensburg, Germany). The codon usage was optimized for *H. jecorina *(http://www.kazusa.or.jp/codon). The synthetic gene *tneub *(for sequence see additional file 1) was similarly obtained based on the protein sequence from *Campylobacter jejuni *NCTC11168 NeuNAc synthase (http://old.genedb.org/genedb/cjejuni/index.jsp, Cj1141).

The synthetic genes *tbage *and *tneub *were excised from the production plasmid using *Xba*I/*Nsi*I digestion and inserted into pRLM_ex_30 [30] to generate the plasmids pMS-PEC and pMS-PSC.

For the construction of pGEX-epi and pGEX-syn, the oligonucleotides GEXfw and GEXrev (Table 1) were used to introduce an *Xba*I and *Nsi*I site into plasmid pGEX4T-2 (GE Healthcare, Chalfont St Giles, UK), yielding pGEX-MS. *tbage *and *tneub *were inserted into pGEX-MS via *Xba*I/*Nsi*I digestion to yield the plasmids pGEX-epi and pGEX-syn.

**Table 1:** Oligonucleotides used during this study

Name	Sequence (5'→3')	Usage
NANASfw	GTGGTGTGCAGGAGGACGAA	qPCR *tneub*
NANASrev	CAAGCACATCGCCCAGTTCAAG	qPCR *tneub*
ManEfw	GCGATCTTGAGCCAGTTCTC	qPCR *tbage*
ManErev	GCTACTTCACCTGCCTCGAC	qPCR *tbage*
GEX-MSfw	AATTCCTTCTAGAGATATGCATC	Construction pGEX-MS
GEX-MSrev	TCGAGATGCATATCTCTAGAAGG	Construction pGEX-MS
pkifw R	CTGCGACACTCAGAACATGTACGT	qPCR *pki *cDNA
pkifw D	GCTCTGCTTGGAACCTGATTGA	qPCR *pki *DNA
pkirev	GGTCTGGTCGTCCTTGATGCT	qPCR *pki*
sar1fw	TGGATCGTCAACTGGTTCTACGA	qPCR *sar1*
sar1rev	GCATGTGTAGCAACGTGGTCTTT	qPCR *sar1*

### Protoplast transformation

The protoplast transformation of *H. jecorina *was performed as described previously [31]. The plasmid pHylox2 (2 μg) [32], which confers hygromycin B resistance [30], and 4 μg of each plasmid pMS-PEC and pMS-PSC were co-transformed into the fungal genome.

### DNA analysis

Fungal genomic DNA was isolated as described previously [31]. Southern hybridization and detection were performed using the DIG High Prime DNA Labeling and Detection Starter Kit II following the manufacturer's instructions (Roche, Basel, Switzerland).

### Transcriptional analysis

RNA extraction, cDNA synthesis and qPCR analysis were performed as described elsewhere [25]. Primer sequences are given in Table 1.

### Glutathione S-transferase (GST) fusion proteins

GST fusion proteins of GlcNAc-2-epimerase and NeuNAc synthase were generated using plasmids pGEX-epi and pGEX-syn in *E. coli *BL21 (DE3). Purification of the proteins was performed using GSTrap™FF (GE Healthcare) according to standard procedures.

### Enzymatic assay

Harvested mycelia were ground into fine powder and resuspended in 0.1 M Bicine buffer (pH 8) containing protease inhibitors (2 μM leupeptin, 1 μM pepstatin A, and 10 μM PMSF) (0.3 g mycelia/mL). The suspension was sonicated using a Sonifier^® ^250 Cell Disruptor (Branson, Danbury, US) (power 40%, duty cycle 50%, power 20 sec, 40 sec pause, 10 cycles). Insoluble compounds were separated using centrifugation (10 min, 13000 g, 4°C). Enzymatic analysis was performed according to a previously described modified protocol [33]. The assay was performed in a total volume of 100 μL containing 10 mM GlcNAc, 10 mM PEP, 12.5 mM MnCl_2_, 100 mM Bicine buffer (pH 8) and 40 μL cell-free extract. Reactions were incubated for 60 min at 37°C, terminated at 85°C for 10 min and analyzed using HPLC. As a positive control, 5 μL of both GST fusion proteins were applied in place of the cell-free extracts.

The stability of NeuNAc in the cell-free extract was determined by adding NeuNAc (150 μM) and incubating for 24 h at 30°C. After derivatization with DMB [34], the NeuNAc quantity was measured using HPLC.

### Detection of NeuNAc synthesis *in vivo*

Harvested *H. jecorina *mycelia were ground into fine powder and resuspended in water (0.3 g mycelia/mL). The suspension was sonicated using a Sonifier^® ^250 Cell Disruptor (Branson) (power 70%, duty cycle 50%, power for 1 min, 1 min pause, 3 cycles). Insoluble compounds were separated using centrifugation (10 min, 13000 g, 4°C), and the supernatant was analyzed using HPLC-MS/MS.

### NeuNAc and GlcNAc uptake

*H. jecorina *mycelia were pre-grown on MA containing 1% glycerol, transferred to MA medium containing 1% glycerol or no carbon source, spiked with 30 μM NeuNAc or GlcNAc, respectively, and incubated for 8 h at 30°C. Autoclaved mycelia served as a negative control. After derivatization with DMB [34], the NeuNAc quantity was measured using HPLC.

### HPLC and HPLC-MS/MS analysis

NeuNAc, ManNAc and GlcNAc formation was measured using LC-MS (IT-TOF-MS) (Shimadzu, Kyoto, Japan) with a Rezex™ RHM-Monosaccharide H^+^-column (8%, 300 × 7.8 mm) (Phenomenex, Torrance, USA). The mobile phase consisted of water with 0.1% (v/v) trifluoroacetic acid, the flow was 0.6 mL/min, the column temperature was 80°C, and the injected volume was 10 μL. MS detection was performed in ESI+ mode, covering a scan range of 60-600 amu. The retention times were determined using pure standard substances. The identity of NeuNAc was confirmed by both, chromatographic retention time and mass spectral signal, which are very well matched by authentic standards of NeuNAc. The better the mass accuracy obtained from exact mass determination by HR-MS, the lower is the number of possible isobaric candidates (*e.g*. [35]). In this case the mass accuracy is better than 2 ppm, leading to the number of candidates reduced to less than 10, with an even further reduction in the number of potential candidates because the isotopic pattern is also taken into account (what the software of the used IT-TOF-MS instrument does automatically).

DMB derivatives of NeuNAc were separated on a Kinetex RP C18 (Phenomenex) at 0.75 mL/min with a 40°C column temperature and a mobile phase of water:methanol:trifluoroacetic acid (74.25:25:0.75). A Shimadzu RF-20AXS fluorescence detector (excitation 373 nm, emission 448 nm) was used for detection.

## Competing interests

The authors declare that they have no competing interests.

## Authors' contributions

MGS produced and characterized the recombinant strains and contributed to the manuscript. ARMA prepared the manuscript and contributed to the design of the study. RG established enzymatic assays. EER performed HPLC-MS/MS analyses; MDM suggested the target substance and supported analytics; RLM contributed to the design and coordination of the study. All authors critically read the manuscript.

## Supplementary Material

Additional file 1 **Coding sequences of the synthetic genes *tbage *and *tneub***. Coding sequences of the synthetic genes *tbage *and *tneub*. The sequences are provided in FASTA format. The ***Xba***I site is underlined, and the ***Nsi***I site is double-underlined. The start codon ATG and the stop codon TAA are presented in bold letters. Click here for file

Additional file 2 **Parameters of *H. jecorina *cultivation on chitin in a bioreactor**. Oxygen consumption (pO2; blue line), consumption of the intermediate N-acetylglucosamine (GlcNAc; red line), formation of the product N-acetylneuraminic acid (NeuNAc; pink line), and formation of biomass (given as dry weight, BDW; green line) of the ***H. jecorina ***PEC/PSC1 strain cultivated on chitin in a bioreactor for 96 h are displayed. Click here for file
