# Supplementary material for: Synthesis of an antiviral drug precursor from chitin using a saprophyte as a whole-cell catalyst
Source: Microb Cell Fact. 2011 Dec 5;10:102. doi: 10.1186/1475-2859-10-102 (PMC3245449; doi:10.1186/1475-2859-10-102)
Supplement: Additional file 1 — Coding sequences of the synthetic genes tbage and tneub. Coding sequences of the synthetic genes tbage and tneub. The sequences are provided in FASTA format. The XbaI site is underlined, and the NsiI site is double-underlined. The start codon ATG and the stop codon TAA are presented in bold letters. [file 1475-2859-10-102-S1.PDF]

Additional file 1. Coding sequences of the synthetic genes *tbage* and *tneub*.

>*tbage*

TCTAGA**ATG**GGCAAGAACCTCCAGGCCCTGGCCCAGCTCTACAAGAACGCCCTC
CTCAACGACGTCCTGCCCTTCTGGGAGAACCACAGCCTCGACAGCGAGGGCGGCT
ACTTCACCTGCCTCGACCGCCAGGGCAAGGTCTACGACACCGACAAGTTCATCTG
GCTCCAGAACCGCCAGGTCTGGACCTTCAGCATGCTCTGCAACCAGCTGGAGAAG
CGCGAGAACTGGCTCAAGATCGCCCGCAACGGCGCCAAGTTCCTCGCCCAGCAC
GGCCGCGACGACGAGGGCAACTGGTACTTTGCCCTGACCCGCGGCGGCGAGCCT
CTGGTCCAGCCCTACAACATCTTCAGCGACTGCTTCGCCGCCATGGCCTTCAGCC
AGTACGCCCTCGCCAGCGGCGAGGAGTGGGCCAAGGACGTCGCCATGCAGGCCT
ACAACAACGTCCTCCGCCGCAAGGACAACCCCAAGGGCAAGTACACCAAGACCT
ACCCCGGCACCCGCCCCATGAAGGCCCTGGCTGTCCCCATGATCCTCGCCAACCT
CACCCTGGAGATGGAGTGGCTCCTCCCCCAGGAGACCCTGGAGAACGTCCTCGCC
GCCACCGTCCAGGAGGTCATGGGCGACTTCCTCGACCAGGAGCAGGGCCTCATGT
ACGAGAACGTCGCCCCCGACGGCAGCCACATCGACTGCTTCGAGGGCCGCCTCAT
CAACCCCGGCCACGGCATCGAGGCCATGTGGTTCATCATGGACATCGCCCGCCGC
AAGAACGACAGCAAGACCATCAACCAGGCCGTCGACGTCGTCCTCAACATCCTC
AACTTCGCCTGGGACAACGAGTACGGCGGCCTCTACTACTTCATGGACGCCGCCG
GCCACCCCCCCCAGCAGCTGGAGTGGGACCAGAAGCTCTGGTGGGTCCACCTGG
AGAGCCTCGTCGCCCTCGCCATGGGCTACCGCCTCACCGGCCGCGACGCCTGCTG
GGCCTGGTATCAGAAGATGCACGACTACAGCTGGCAGCACTTCGCCGACCCTGA
GTACGGCGAGTGGTTCGGCTACCTCAACCGCCGAGGCGAGGTCCTCCTCAACCTC
AAGGGCGGCAAGTGGAAGGGCTGCTTCCACGTCCCCCGCGCCATGTACCTCTGCT
GGCAGCAGTTCGAGGCCCTCAGC**TAA**TGCAT

>*tneub*

TCTAGA**ATG**CAGATCAAGATCGACAAGCTCACCATCAGCCAGAAGAACCCCCTC
ATCATCCCCGAGATCGGCATCAACCACAACGGCAGCCTGGAGATCGCCAAGCTC
ATGGTCGACGCCGCCAAGCGAGCCGGCGCCAAGATCATCAAGCACCAGACCCAC
ATCGTCGAGGACGAGATGAGCCAGGAGGCCAAGAACGTCATCCCCGGCAACGCC
AACATCAGCATCTACGAGATCATGGAGCAGTGCGCCCTCAACTACAAGGACGAG
CTGGCCCTCAAGGAGTACGTCGAGAAGCAGGGCCTCGTCTACCTCAGCACCCCCT
TCAGCCGCGCCGCCGCCAACCGCCTGGAGGACATGGGCGTCAGCGCCTACAAGA
TCGGCAGCGGCGAGTGCAACAACTACCCCCTGATCAAGCACATCGCCCAGTTCAA
GAAGCCCATGATCATCAGCACCGGCATGAACAGCATCGAGAGCATCAAGCCCAC
CGTCAAGATCCTCCGCGACTACGAGATCCCCTTCGTCCTCCTGCACACCACCAAC
CTCTACCCCACCCCCAGCCACCTCGTCCGCCTCCAGGCCATGCTGGAGCTGTACA
AGGAGTTCAACTGCCTCTACGGCCTCAGCGACCACACGACGAACAACCTCGCCTG
CATCGGCGCCATCGCCCTCGGCGCCAGCGTCCTGGAGCGCCACTTCACCGACACC
ATGGACCGCAAGGGCCCCGACATCGTCTGCAGCATGGACGAGAGCACCCTCAAG
GACCTCATCAACCAGACCCAGGAGATGGTCCTCCTCCGCGGCGACAACAACAAG
AACCCCCTGAAGGAGGAGCAGGTCACCATCGACTTCGCCTTCGCCAGCGTCGTCA
GCATCAAGGACATCAAGAAGGGCGAGATCCTCAGCATGGACAACATCTGGGTCA
AGCGCCCCAGCAAGGGCGGCATCAGCGCCAAGGACTTCGAGGCCATCCTCGGCA
AGCGCGCCAAGAAGGACATCAAGAACAACATCCAGCTCACCTGGGACGACTTCG
AG**TAA**TGCAT
