# Supplementary figures and images for: Synthesis of an antiviral drug precursor from chitin using a saprophyte as a whole-cell catalyst
Source: Microb Cell Fact. 2011 Dec 5;10:102. doi: 10.1186/1475-2859-10-102 (PMC3245449; doi:10.1186/1475-2859-10-102)

**Additional file 2.** Parameters of *H. jecorina* cultivation on chitin in a bioreactor.

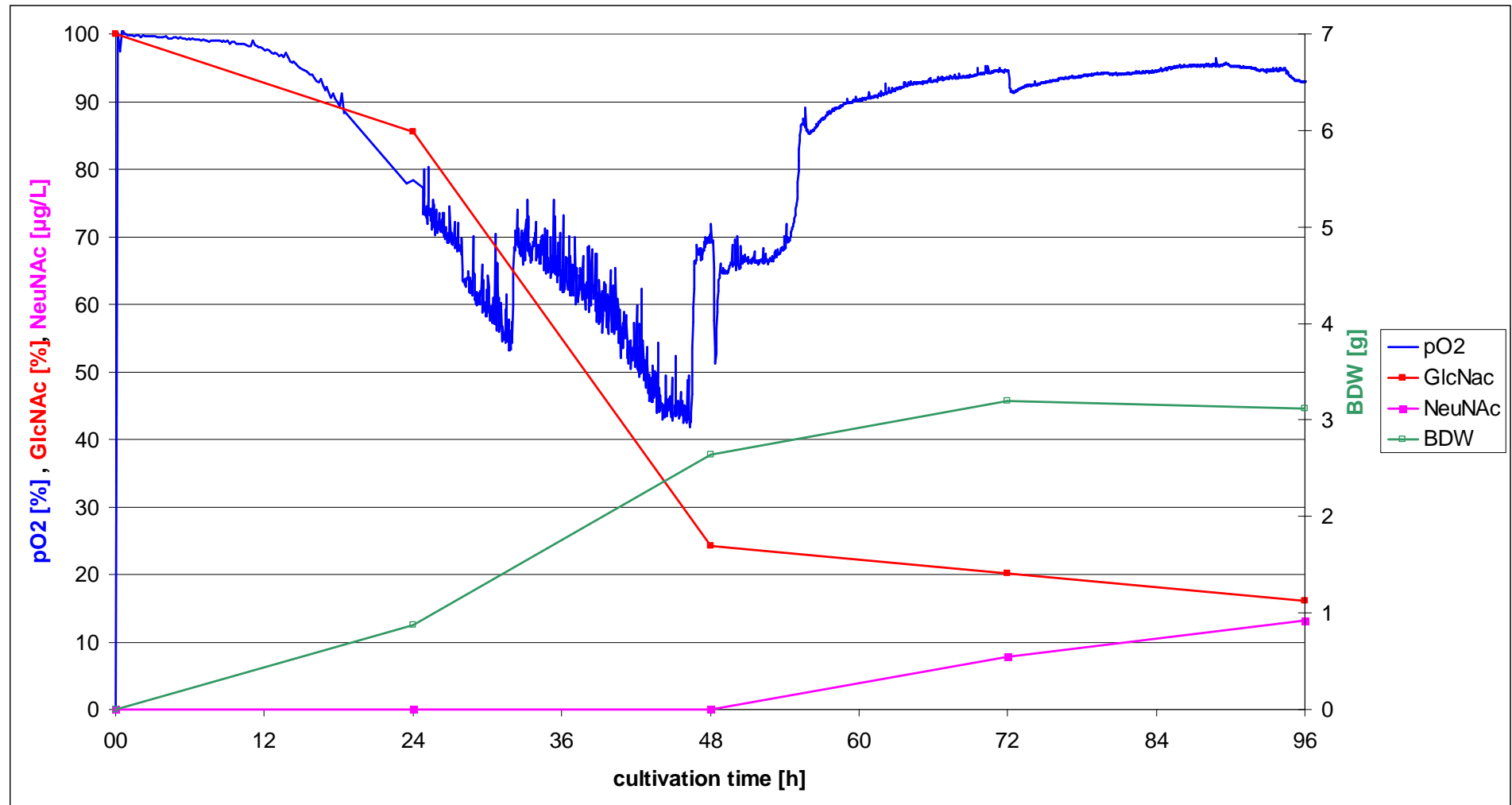

Supplement: Additional file 2 — Parameters of H. jecorina cultivation on chitin in a bioreactor. Oxygen consumption (pO2; blue line), consumption of the intermediate N-acetylglucosamine (GlcNAc; red line), formation of the product N-acetylneuraminic acid (NeuNAc; pink line), and formation of biomass (given as dry weight, BDW; green line) of the H. jecorina PEC/PSC1 strain cultivated on chitin in a bioreactor for 96 h are displayed. [file 1475-2859-10-102-S2.PDF]
